# Processing of fMRI-related anxiety and information flow between brain and body revealed a preponderance of oscillations at 0.15/0.16 Hz

**DOI:** 10.1038/s41598-022-13229-7

**Published:** 2022-06-01

**Authors:** Gert Pfurtscheller, Katarzyna J. Blinowska, Maciej Kaminski, Beate Rassler, Wolfgang Klimesch

**Affiliations:** 1 Institute of Neural Engineering, Graz University of Technology, Graz, Austria; 2 Nalecz Institute of Biocybernetics and Biomedical Engineering, Polish Academy of Sciences, Ks. Trojdena 4 St., 02-109 Warsaw, Poland; 3 Faculty of Physics, University of Warsaw, Ul. Pasteura 5, 02-093 Warsaw, Poland; 4 Carl-Ludwig-Institute of Physiology, University of Leipzig, Leipzig, Germany; 5 Centre of Cognitive Neuroscience, University of Salzburg, Salzburg, Austria

**Keywords:** Neural circuits, Neuro-vascular interactions

## Abstract

Slow oscillations of different center frequencies and their coupling play an important role in brain-body interactions. The crucial question analyzed by us is, whether the low frequency (LF) band (0.05–0.15 Hz) or the intermediate frequency (IMF) band (0.1–0.2 Hz) is more eminent in respect of the information flow between body (heart rate and respiration) and BOLD signals in cortex and brainstem. A recently published study with the LF band in fMRI-naïve subjects revealed an intensive information flow from the cortex to the brainstem and a weaker flow from the brainstem to the cortex. The comparison of both bands revealed a significant information flow from the middle frontal gyrus (MFG) to the precentral gyrus (PCG) and from brainstem to PCG only in the IMF band. This pattern of directed coupling between slow oscillations in the cortex and brainstem not only supports the existence of a pacemaker-like structure in brainstem, but provides first evidence that oscillations centered at 0.15/0.16 Hz can also emerge in brain networks. BOLD oscillations in resting states are dominating at ~ 0.08 Hz and respiratory rates at ~ 0.32 Hz. Therefore, the frequency component at ~ 0.16 Hz (doubling-halving 0.08 Hz or 0.32 Hz) is of special interest, because phase coupled oscillations can reduce the energy demand.

## Introduction

In a recently published paper^1^ we reported for the first time on directed information flow (directed coupling, based on the Granger causality principle^2^) between blood-oxygenation-level-dependent (BOLD) signals from the prefrontal cortex (PFC) and brainstem during the decline of initially elevated MRI-related anxiety. Significant differences in the low frequency (LF) band (0.05–0.15 Hz) were found between elevated and low/no anxiety in the downward information flow from middle frontal gyrus (MFG) to pontine structures in brainstem and in the ascending flow from brainstem to precentral gyrus (PCG) in healthy MRI-naïve participants. The ascending flow provides support for a central mechanism in form of an autonomous central oscillator (pacemaker) in the brainstem^3–8^. The PCG is not only part of the large-scale sensorimotor brain network which plays an important role in anxiety disorders^9^, but is also important in interoceptive perception^10^. Keller et al. ^10^ studied neural correlates of fluctuations in the LF (0.05–0.12 Hz) band and intermediate frequency (IMF: 0.12–0.18 Hz) band between heart rate variability (HRV) power and BOLD signals related to interoceptive perception and reported on a higher correlation of these signals in the IMF in comparison to the LF band. The largest correlation was found between HRV power and BOLD signals in the insula and left PFC. These findings underline the importance of oscillations centered around 0.15 Hz^11^ and the importance of the PFC and pontine structures in emotional processing^12^. One important limitation of our first causality study^1^ was the use of the standardized LF band (Task Force^13^) with an upper cutoff frequency of 0.15 Hz, another limitation was to study only fMRI participants with successful anxiety processing.

**Figure 1:**
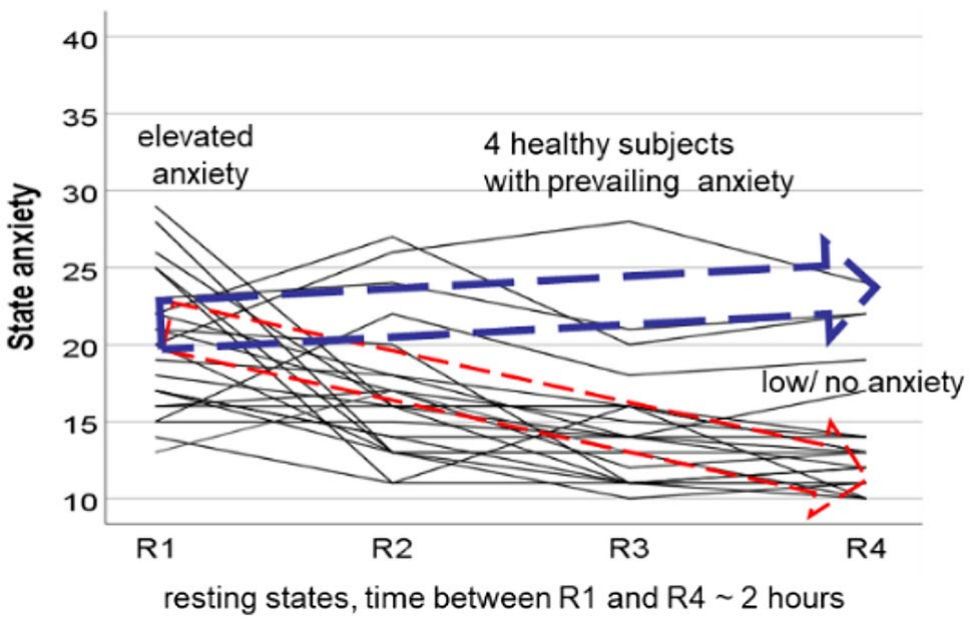
Trajectories of the state anxiety in 23 healthy MRI-naïve young subjects across different periods of resting states. The red arrow with stippled lines indicates the sample of 14 subjects with successful anxiety processing. The blue arrow marks four healthy subjects with prevailing anxiety. Horizontal axis: resting states R1 to R4, vertical axis: state anxiety from AS = 10 (no anxiety) to 40 (maximal anxiety). Modified by^1^.

In the current paper we survey slow spontaneous oscillations in the IMF (0.1–0.2 Hz) band and compared the results with the standard LF (0.05–0.15 Hz) band with the focus on prevailing fMRI-related anxiety in healthy participants. Additionally, in the sample of participants with successful anxiety processing (declining anxiety) the group with low/no anxiety was studied. The decline or habituation of anxiety is not found in all subjects. Chapman^14^ already reported that in a small cohort of healthy MRI-naïve young men anxiety increased again toward the end of scanning sessions. In Fig. 1 (modification of Fig. 1 from^1^), the trajectories of state anxiety (AS) of our data pool consisting of 23 healthy fMRI-naïve young participants demonstrate that the majority of participants showed the expected anxiety decline. However, in a few healthy participants anxiety exhibited an increase. This is a remarkable observation, which raises the important question, whether some processes initiated by the novel experience of the noisy, constricted space within the scanner may act as precondition for a possible break-off of the fMRI examination. The nearly 2% of MRI abortions in patients due to claustrophobia^15^ is a high percentage, particular when the high number of psychiatric disorders^16^ is considered.

One aim of the study was to compare the information flow in the LF and IMF (0.1–0.2 Hz) bands between cortex and brainstem for low and elevated anxiety. If an autonomous oscillator (pacemaker) is located in the brainstem, a strong outflow to the cortex in the IMF band is expected to be observed during elevated anxiety. Another aim was to find out whether slow BOLD oscillations with different origins in the brainstem can be differentiated, with a special focus on the cardiac beat-to-beat interval (RRI: interval between two consecutive R waves) signal. Finally, we asked whether it is possible to identify oscillations centered at ~ 0.15 Hz with an origin in cortical or subcortical structures.

## Methods

### Study approval

All participants gave informed written consent to the protocol of the study, which was approved by the local Ethics Committee at the University of Graz (number: GZ. 39/75/63 ex 2013/14). Our research was performed in accordance with the ethical standards laid down in the 1964 Declaration of Helsinki.

### Study design

A prerequisite to study anxiety processing during scanning in healthy fMRI participants is to define groups with high anxiety (HA) and with low or no anxiety (LA). The base for this was a fMRI study with four resting states, respiration and ECG recording during scanning, and within-scanner questionnaire^1, 17, 18^.

### Experimental paradigm

The experimental design consisted of four resting states, two (R1, R2) in the first session (lasting about 45 min) and two (R3, R4) in the second session (same duration as session 1). For measuring state anxiety, within-scanner questionnaires were carried out just before the resting states R1 and R3 and just after the resting states R2 and R4. Each resting state lasted about 350 s, filling in each questionnaire took approximately 5 min. State anxiety (AS) was assessed with the state-trait anxiety and depression inventory (STADI^19^)**.** The items were presented on a screen within the scanner. Items were answered via a trackball. For further details see^1^.

### Participants

A total of N = 23 participants (12 female, 22 right-handed) between 19 and 34 years of age (M = 24, SD = 3.2 years) took part in the fMRI study. Participants were naïve to the purpose of the study, had no former MRI experience and were without any record of neurological or psychiatric disorders (assessed by self-report). All participants gave informed written consent to the protocol of the study, which had been approved by the local Ethics Committee at the University of Graz.

The majority of 23 participants displayed two clear patterns, a decline of anxiety from the first to the last resting states, pointing to a successful anxiety processing in 14 participants and a prevailing or increasing anxiety in 4 participants. In five participants no clear pattern was found. In one person the first scanning sequence (R1) was equal with the second sequence (R2) due to a technical mistake, and in four persons ECG recordings were corrupted by strong MR-gradient artefacts. Therefore, these participants were excluded from further analysis.

In our first study^1^ successful processing of anxiety was studied in the sample of 14 participants. From each participant in this sample two measurements were available, one from the state with the highest AS score (mostly R1) and another with the lowest AS score (mostly R4). The first measurement with elevated anxiety was leveled HA14 and the other measurement with low/no anxiety was leveled LA14. In these two groups (HA14, LA14) no differentiation between vascular and neural BOLD signals was made.

Four out of 23 healthy participants (~ 20%) not only exhibited prevailing anxiety in the last resting states (R3, R4), but also a dominance of neural BOLD components with RRI preceding BOLD waves. The study of only these 4 participants is statistically not meaningful, therefore we formed a group of 11 persons (HA11), embedding also these 4 persons, all with elevated anxiety (AS > 15) and dominant neural BOLD oscillations at least in one resting state. In the groups LA14 (AS = 12.5 ± 1.5) and HA11 (AS = 20.2 ± 3.3) the information flow in the LF and IMF bands was investigated.

### Physiological signal recording and RRI time courses

Electrocardiogram (ECG) and respiration were recorded inside the scanner. The sampling rate was 400 Hz. QRS detection and subsequent computation of RRI time series were performed using fMRI plug-in for EEGLAB^20^. To further improve RRI signals, the Kubios HRV Premium Package^21^ was used. For further details see^1^.

### Resting state fMRI and ROI selection

Functional images were acquired with a 3 T scanner (Magneton Skyra, Siemens, Erlangen, Germany) using a multiband GE-EPI sequence^22^ with a simultaneous six-band acquisition with TE/TR = 34/871 ms, 52° flip angle, 2 × 2 × 2 mm^3^ voxel size, 66 contiguous axial slices (11 × 6), acquisition matrix of 90 × 104 and a FOV of 180 × 208 mm^2^. Finally, the AAL^23^ atlas was used to extract time courses for specified regions of interest (ROIs) in the left pre-central gyrus (PCG, ROI 1), left middle frontal gyrus (MFG, ROI 7) and left cerebellum/ brainstem (ROI 93, ROI 103). For the analysis epochs of 53 s were used. For further details see^8^.

Important is the disentangling of respiration-related BOLD artefacts from neural BOLD oscillations and the discussion of aliasing in connection with heart rate-related movements in brainstem. The study of BOLD signals in 18 ROIs in brainstem revealed significant side-differences with a dominance of respiratory BOLD artefacts in the right side and neural BOLD signals in the left side^8^. This was the reason to do without any retrospective correction method and only to study BOLD signals in the left brainstem. Movements of blood pressure in brainstem at a rate of e.g. 60 beats/min (corresponds to 1 Hz) results in an aliasing frequency at ~ 0.15 Hz with a scanning rate of TR = 0.871 s (corresponds to 1.15 Hz). The checking of both, breathing (sampled at 400 Hz) and BOLD signals around 0.15 Hz, revealed a perfect concordance even with varying frequencies, therewith an aliasing effect can be excluded.

### Computing of causal coupling and statistic

The interaction between the time series was calculated by means of Directed Transfer Function (DTF^24^), which allows to estimate causal coupling between the signals as a function of frequency. DTF is based on the Granger causality principle^25^, which states that for two time series, if the variance of the prediction error for the second time series is reduced by including past measurements from the first time series in the linear regression model, then the first time series can be said to cause the second time series. Granger causality principle is equivalent to 2-channel Multivariate Autoregressive Model (MVAR). The Granger principle may be extended to arbitrary number of channels^2^ by considering MVAR for *k* channels (*k* > 2).

We can express the MVAR model for *k* channels in the form:

1 $${\mathbf{X}}(t) = \sum\limits_{j = 1}^{p} {{\mathbf{A}}(j){\mathbf{X}}(t - j) + {\mathbf{E}}(t)}$$

where ${\mathbf{X}}(t) = (X_{1} (t), \, X_{2} (t), \, \ldots , \, X_{k} (t))^{{\text{T}}}$ is a vector of signals, **A**(*j*) is a *k*x*k* matrix of model coefficients, **E**(*t*) is a *k*-size vector of white noises, *p*—model order (the number of samples we take into account in the regression). The MVAR model assumes that *X*_1_(*t*)—a sample of signal at a time *t*—can be expressed as a sum of *p* previous values of signal *X*_1_(*t*) and also other signals of the **X**(*t*) vector weighted by model coefficients **A**(*j*) plus a random noise **E**(*t*).

By fitting *k* channels time series to the model we get the model coefficients **A**(*j*). By transforming both sides of Eq. (1) to the frequency domain by the Z transform, which is customary used for discrete signals, we get:

2 $${\mathbf{X}}\left( z \right) \, = {\mathbf{H}}\left( z \right){\mathbf{E}}\left( z \right),$$

Transformed functions are denoted by capital letters, *z*^-1^ is a unit delay operator in the form: z^−1^ = exp(− *i*2π*f*∆*t*), *m* is a number of determined model coefficients, **H**(*z*) is a transfer matrix of the MVAR model of the dimension *k*x*k*

3 $$H(f) = \left( {\sum\limits_{m = 0}^{p} {A(m)\exp ( - 2\pi imf\Delta t)} } \right)^{ - 1}$$

The elements *H*_*ij*_ of matrix **H**(*f*) describe the connection between the *j*-th input and *i*-th output of the system and they are measures of the directed information flow.

DTF is formulated in terms of matrix **H**(*f*) elements^24^.

4 $${\text{DTF}}_{ij} (f) = \frac{{\left| {H_{ij} (f)} \right|^{2} }}{{\sum\limits_{m = 1}^{k} {\left| {H_{im} (f)} \right|^{2} } }}.$$

DTF_*ij*_(*f*) describes causal influence of signal *j* on signal *i* at frequency *f* normalized in respect of inflows to the destination channel *i*. DTF_*ij*_(*f*) takes values in the [0–1] range.

Herein the version of DTF, called ffDTF, was used:

5 $${\text{ffDTF}}_{ij} (f) = \frac{{\left| {H_{ij} (f)} \right|^{2} }}{{\sum\limits_{f} {\sum\limits_{m = 1}^{k} {\left| {H_{im} (f)} \right|^{2} } } }},$$

ffDTF differs from DTF by the normalization factor in the denominator of Eq. (1), which involves integration over frequencies.

ffDTF is a function of frequency, so in order to quantitatively estimate the strength of coupling, the ffDTFs were integrated in the bands of interest, namely: LF (0.05–0.15 Hz) and IMF (0.1–0.2 Hz) and were averaged over epochs. In this way *C*_*ij*_, the coupling strength between signals *j* and *i* in the selected frequency band (obtained from ffDTF), was found.

Significant differences between coupling values involving the directions of the information flows between channels were found by means of a bootstrap method described in^1^. The significance of the differences between inflows and outflows from/to the given channel were determined as percentiles of the distribution obtained by bootstrap. Those differences lying outside the confidence range of 95% were considered as significant.

## Results

The two groups studied revealed slightly different heart rates (HR) and respiratory frequencies (RF) for low/no anxiety (LA14) and elevated anxiety (HA11) both frequencies being higher for elevated anxiety: low/no anxiety: HR = 60.9 ± 8.4 beats/min, RF = 0.26 ± 0.06 Hz; elevated anxiety: HR = 63.8 ± 9.3 beats/min and RF = 0.33 ± 0.08 Hz. During elevated anxiety three subjects displayed coherent slow RRI and breathing oscillations at ~ 0.1 Hz. These three subjects are not included in the calculation of mean respiratory frequency.

Slow BOLD oscillations either precede or lag RRI signals. The former case is characteristic for vascular BOLD, the latter case for neural BOLD oscillations whereby the time difference between vascular and neural BOLD components is ~ 2.5 s^17, 26^. The selected 11 subjects from the HA11 group displayed a significant (*p* < 0.05) positive time delay (pTD) of 1.5 s ± 0.7 (mean ± SD) in the brainstem for ROI 103 and of 1.7 s ± 0.8 for ROI 93. The corresponding significant bins (samples of time series) varied around 36%^17^. This number indicates the duration of significant phase coupling between BOLD and RRI signals.

### Causal coupling during low/no anxiety in LF and IMF bands

**Figure 2:**
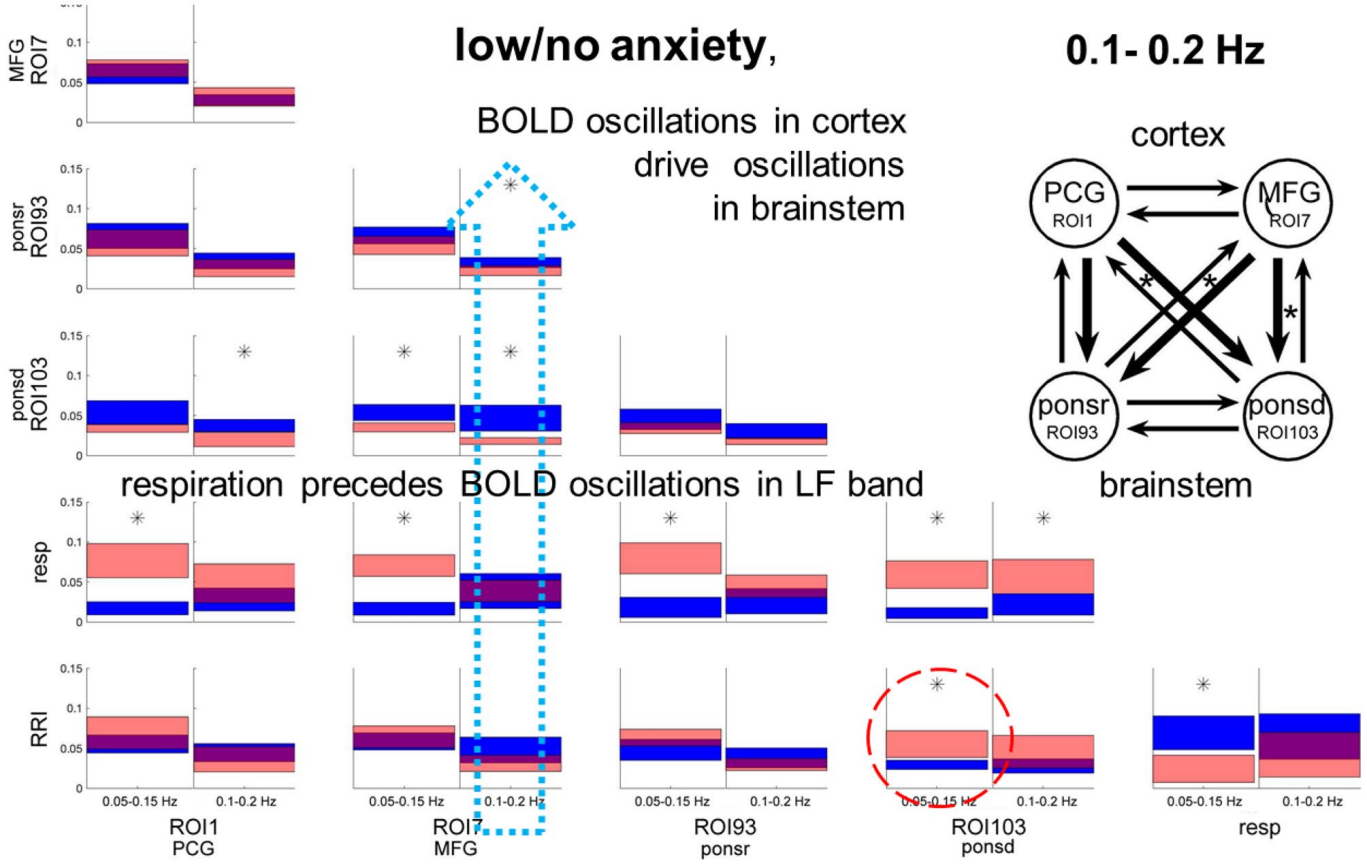
Directed coupling strengths for the low/no (LA) anxiety group in the IMF band (right columns) and the LF band (left columns). Each box shows the strength of coupling as a position on the vertical axis. The width of bars is proportional to the mean error (errors were based on percentiles of distribution). The blue color shows the flow from the signal marked below the given column to the signal marked on the left, and the red color the flow from the signal marked on the left to the signal marked below. Significant differences between couplings of in- and outflow are marked by stars (*p* < 0.05). Note the significant information flows from the MFG (ROI 7) to the pons (ROI 93,103), RRI and respiration, which is indicated by a stippled blue arrow. In addition, there is a significant information flow from RRI signals to the brainstem in the LF band (marked by a stippled circle). The upper insert shows the directed coupling strengths between BOLD signals only, encoded by the thickness of the arrows. Each arrow indicates the direction and the strength of information flow, stars mark significant changes (*p* < 0.05).

The strength of the information flow between structures was based on causal coupling values calculated as spectral power of ffDTF functions in selected frequency bands averaged over subjects. The information flow differs between the IMF band (0.1–0.2 Hz; Fig. 2 right columns) and the LF band (0.05–0.15 Hz; Fig. 2 left columns). In the former case three ROI pairs represent significant differences in flows between BOLD signals (indicated by stars), in the latter only one pair. The most significant differences are the outflow from the MFG (ROI 7) and the inflow into the brainstem (ROI 103) in the 0.1–0.2 Hz band. In contrast, the flow from respiration to BOLD signals is highly significant in the LF band. It is worth mentioning that the flow from RRI to BOLD signals in the brainstem (ROI 103) in the LF band also reached significance. The strength of flows and their significance (marked by stars) between the four BOLD signals are indicated in the insert. The statistical results for both frequency bands are summarized in Table 1.

**Table 1:** Significance of differences of flows from structures marked above the columns to the ones marked at the left of the rows.

	ROI 1	ROI 7	ROI 93	ROI 103	Resp.
**0.05–0.15 Hz**					
ROI 7	79.02				
ROI 93	23.69	6.12			
ROI 103	2.93	**0.44**	7.07		
Resp.	**99.96**	**100.00**	**99.99**	**99.95**	
RRI	83.93	72.81	96.02	99.25	**0.36**
**0.1–0.2 Hz**					
ROI 7	61.23				
ROI 93	10.01	**1.50**			
ROI 103	**0.61**	**0.51**	2.83		
Resp.	89.74	54.62	96.33	**99.14**	
RRI	18.04	5.99	16.12	91.82	14.33

The numbers show percentiles of the distributions obtained by the bootstrap approach^1^ for low/no anxiety group in the LF band (upper part) and the IMF band (lower part). Significant differences (marked by bold numbers) are those, which exceed the range of 95% of the random distribution of the differences obtained by the bootstrap method. Depending on the sign of the differences between in- and outflows, they are located below 2.5 and above 97.5 percentile.

### Causal coupling during prevailing anxiety in LF and IMF bands

For BOLD signals, significant information flows were observed only in the IMF band from MFG (ROI 7) to PCG (ROI 1) and from the brainstem (ROI 93) to PCG. These results mean that during elevated anxiety the main information flow (and its processing capacity) is restricted to the IMF band. Worth mentioning are the significant flows from the brainstem (ROI 93) and the MFG to the RRI signal in the IMF band during elevated anxiety. The statistical results for both frequency bands are summarized in Table 2.

**Table 2:** Differences between in- and outflows in terms of percentiles of the distribution obtained by the bootstrap approach for the HA11 group.

	ROI 1	ROI 7	ROI 93	ROI 103	Resp.
**0.05–0.15 Hz**					
ROI 7	92.17				
ROI 93	90.98	12.37			
ROI 103	93.61	26.00	67.80		
Resp.	**99.81**	**99.86**	**99.27**	97.43	
RRI	60.76	8.22	9.69	6.44	**1.04**
**0.1–0.2 Hz**					
ROI 7	**97.99**				
ROI 93	**98.00**	16.67			
ROI 103	76.28	6.21	21.16		
Resp.	**98.41**	84.42	79.16	94.99	
RRI	8.70	**1.35**	**0.77**	12.85	**2.39**

For further explanation, see Table 1.

### Discrimination between different types of BOLD oscillations in brainstem

We recorded three types of signals: RRI from thorax, respiration with chest belt and BOLD oscillations. The cardiac function is controlled by the cardiovascular center in the lower brainstem, and respiration by two respiratory centers in the upper and lower brainstem^7, 27^. These centers consist of a mass of neurons and are associated with hemodynamic responses in form of slow BOLD oscillations. BOLD signals in the brainstem were recorded in axial slices of pontine structures in intervals of 2 mm. Because of the small size of the brainstem, the quality of the BOLD signals varied across the different ROIs, with best results for ROIs 93 and 103 (details see^8^). The two ROIs 93 and 103 located in pons are segregated by 10 mm, the latter more caudal and closer to the cardiovascular and the two respiratory centers. Activation of a bulk of neurons in these centers results in BOLD responses that are best recordable in ROI 103 (Fig. 2).

**Figure 3:**
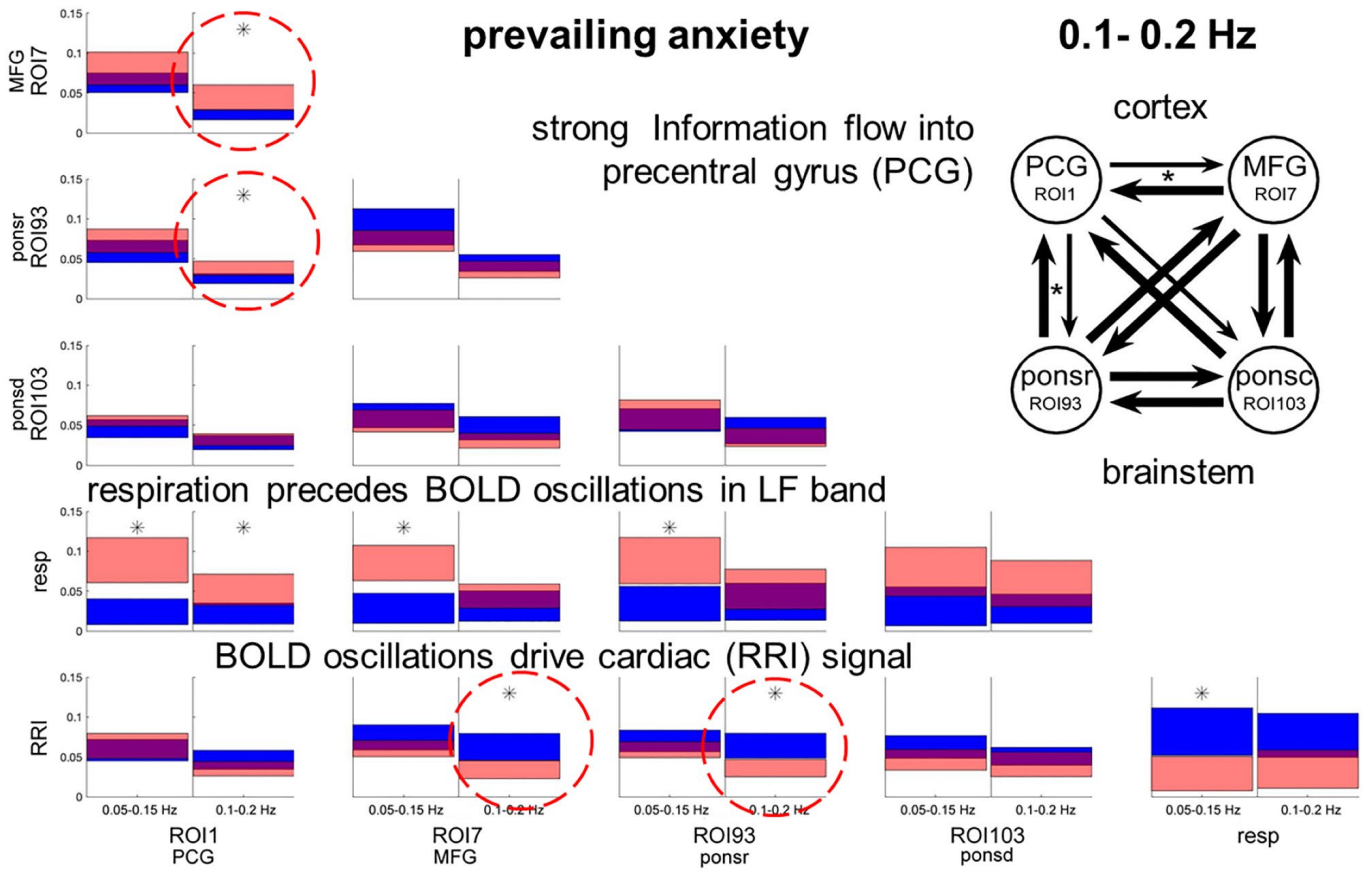
Directed coupling strengths for prevailing anxiety (HA11 group) in the LF and IMF bands. Note the significant flows from MFG (ROI 7) to PCG (ROI 1) and from rostral pons structures (ROI 93) to the PCG (ROI 1) (marked by stippled circles). Also of interest are the significant flows from the MFG and the brainstem (ROI 93) to RRI (marked by stippled circles). For further explanation see Fig. 2.

**Figure 4:**
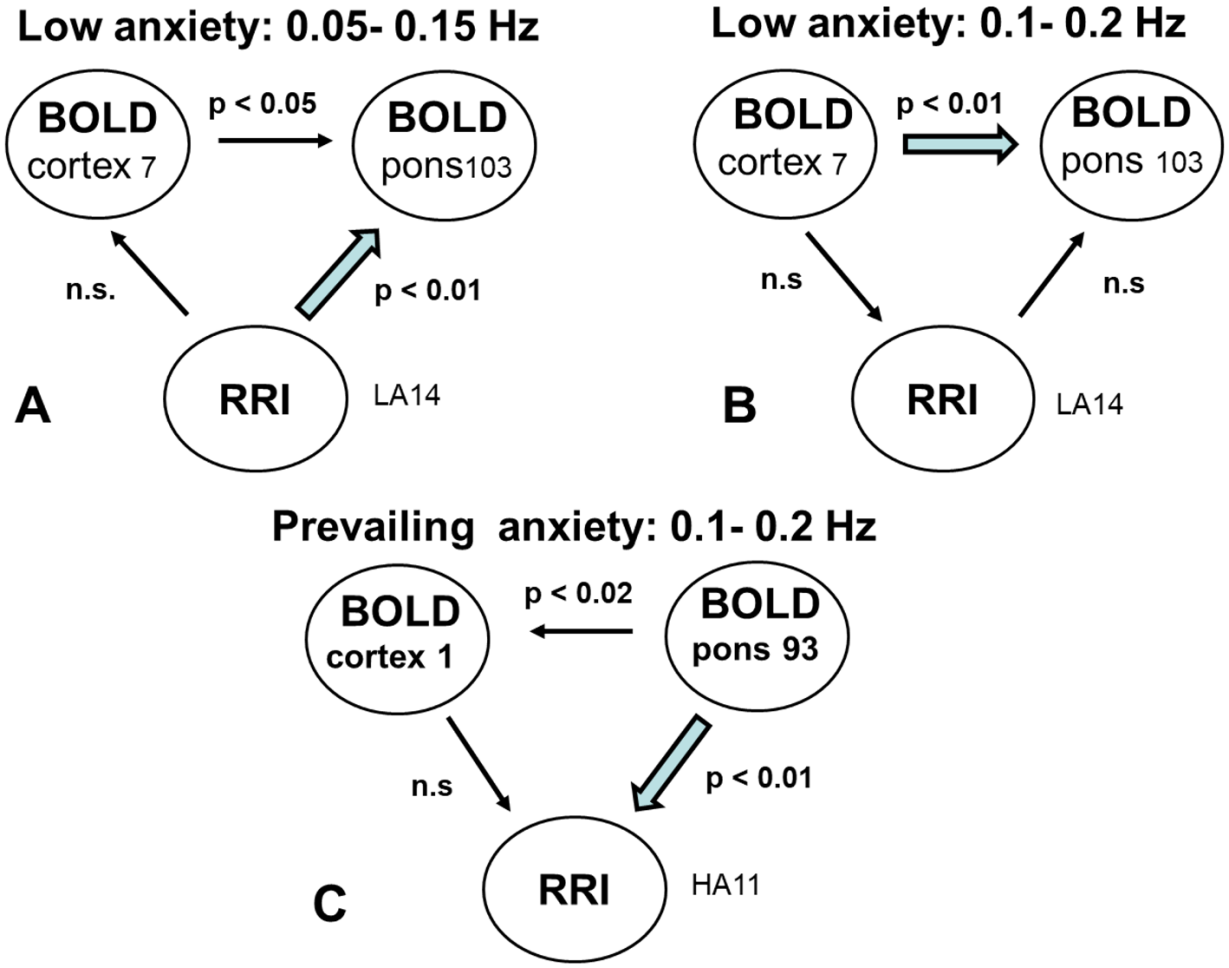
Diagrams for directed coupling between slow BOLD oscillations (in the cortex and the brainstem) and RRI signals. (**A**) Significant information flow during low/no anxiety from cortex and RRI to the brainstem in the LF band. (**B**) Significant information flow during low/no anxiety from cortex to the brainstem in the IMF band. (**C**) Dominant information flow during prevailing anxiety from brainstem to RRI and into cortex in the IMF band. Blue arrows indicate the most important information flows.

As expected, the main findings are the most dominant frequencies in the LF band for RRI and respiration with associated BOLD response in brainstem and less dominant frequencies in the IMF band (Fig. 2). The relationship between RRI and BOLD signals for low/no anxiety is shown for the LF band in Fig. 4A and for the IMF band in Fig. 4B. One novel finding is the strong information flow from the cortex (MFG, ROI 7) to the brainstem in the group with low anxiety (Figs. 2 and 4B) and the flow from brainstem to cortex (PCG, ROI 1) and to RRI in the group with elevated anxiety (Figs. 3 and 4C). Both flows are dominant in the IMF band. It can be speculated, that the significant flow during elevated anxiety in the IMF band with origin in the brainstem (Fig. 4C) may act as driving force for the 0.1–0.2 Hz oscillations in RRI signal.

Here the question arises, why, on the one hand, slow oscillations in RRI signals (LF band) precede BOLD oscillations in brainstem (Fig. 4A), and, on the other hand, a cortical source or oscillator in brainstem acts as driving force for RRI signals in the IMF band (Fig. 4C). An answer could be, that beside the two physiological signals RRI and respiration with associated BOLD response, a third signal in form of a pacemaker in brainstem becomes active during anxiety. This rhythmic neural source (pacemaker) operating in the frequency range 0.1–0.2 Hz can’t be assessed directly, however, may be traced in the recording of the associated BOLD response.

## Discussion

For the discussion of our findings, a few physiological facts have to be emphasized: (i) Neural activity is always accompanied by a hemodynamic response known as neurovascular or neurometabolic coupling^28–31^. The information flow is from neural to BOLD signals with a time delay of 2–3 s. (ii) In a normal resting state without anxiety and stress, inspiration is accompanied by a HR acceleration (decrease of RRI) and expiration by a HR deceleration (increase of RRI), both characteristic for respiratory sinus arrhythmia (RSA)^32^. The dominant information flow in this case is from respiratory to cardiac signals. Recent research from Rassler et al.^33, 34^ shows that besides the classical or positive RSA also a paradoxical beat-to-beat interval (RRI) increase (cardio-deceleration) during inspiration or negative RSA (nRSA) can be observed^35^. In this case, the interaction between respiration and RRI is dominated by a strong feedback with a preference for a flow from cardiac to respiratory signals. (iii) An important principle of living systems is to minimize the energy demand. This can be achieved when different systems or physiological rhythms become harmonically phase synchronized. Klimesch^36, 37^ introduced the doubling-halving algorithm, which describes the frequency architecture of EEG rhythms during conscious cognition, which are coupled to body oscillations such as heart rate and breathing. Center frequencies are 1.25 Hz for HR and 0.63 Hz for breathing muscles. For breathing, there are three center frequencies, one at 0.16 Hz, another at 0.33 Hz (breathing waves) and yet another at 0.08 Hz (slow blood pressure waves). One simple way to document phase synchronization between cardiovascular, respiratory and neural systems is stimulus-paced, conscious breathing at the resonance frequency of the baroreflex loop at ~ 6 breaths/min^38, 39^. Here the question raises, whether such a harmonic phase synchronization is also due to an autonomous (unconscious) process. Note, that in the case of resonance, no clear direction of information flow is defined.

Another interesting observation is that nasal breathing at 0.16–0.33 Hz entrains limbic oscillations in the piriform human cortex, amygdala and hippocampus to the human respiratory cycle^40, 41^. Notably, this entrainment was markedly diminished during oral breathing. With nasal but not with oral breathing, emotional discrimination of fear was accelerated in a respiratory-phase-dependent manner^40^. Thus, we speculate that nasal breathing at ~ 0.16 Hz may have a positive effect to suppress elevated fMRI-induced anxiety. These observations are well in agreement with the binary hierarchy theory of brain and body oscillations^37^.

### Downward information flow from cortex to brainstem during low/no anxiety

The results of low anxiety (LA14 group) for the LF band revealed the expected dominant information flow from respiration to BOLD signals and from RRI to BOLD signals in the LF band (Fig. 2), more significant for respiratory than for cardiac signals. The LA14 group was composed by 14 healthy subjects with 13 persons displaying low and only one subject no anxiety (mean AS = 12.5; the AS scale is from 10 to 40). Thus, the group represents a resting state with low anxiety (AS < 15) but, nevertheless can be considered as a rough approximation of a resting state without anxiety and stress. Characteristic for this group was the prominent information flow from MFG to brainstem (ROIs 93, 103) and from PFC to brainstem (ROI 93) in the 0.1–0.2 Hz band. The flow was most pronounced from MFG to brainstem (ROI 103) and emphasizes therewith a source of rhythms centered at 0.15 Hz in brain networks at least during low anxiety.

From above it follows that the predominance of slow oscillations (0.1–0.2 Hz) in BOLD signals is not restricted to elevated anxiety, but is also found during low/no anxiety. This is not surprising, because one of the first reports on “cardiovascular rhythms in the 0.15-Hz band” was made under normal laboratory situations^11^. This means that such a slow rhythm with an origin in the reticular formation in the brainstem^42^ is a normal physiological phenomenon found in blood pressure waves and RRI signals. A comprehensive study on RRI and systolic arterial blood pressure (BP) signals in the LF band revealed two principal frequency components, one at 0.076 Hz ± 0.012 (mean ± SD) and another at 0.117 ± 0.016 Hz^43^. Due to the limitation of frequencies below 0.15 Hz (part of study protocol) the frequency component at 0.117 Hz was very likely truncated. The fact, however, is that BP oscillations below 0.1 Hz (centered at 0.08 Hz) and above 0.1 Hz can be differentiated. Support for BOLD oscillations in the frequency range of 0.06–0.09 Hz, peaking at 0.08 Hz and in the frequency range of 0.13–0.17 Hz, peaking at 0.15 Hz came from a functional connectivity resting state study^44^. The frequency components centered at 0.08 Hz and between 0.13 and 0.17 Hz have been associated with low frequency vascular oscillations^45, 46^, whereby the frequencies below 0.1 Hz revealed robust network patterns^47^. The effect of vasomotion may be embodied in the 0.13–0.17 Hz band. The origin of vasomotion observed in fMRI could be caused by oscillations in both vascular diameter and blood oxygen supply^47, 48^,. Concerning vasomotion it is important to note, that entrainment of arteriole vasomotor fluctuations by neural activity is a basis for BOLD resting state connectivity^49, 50^. We therefore speculate that BOLD oscillations peaking at 0.08 Hz are related to slow cerebral blood flow circulation with an origin in the baroreflex loop^46, 51^, and BOLD oscillations peaking between 0.13 and 0.17 Hz are of neural origin. This neural aspect of the oscillations in the range 0.13–0.17 Hz is supported by the slow neural correlation fluctuations in the IMF (0.12–0.18 Hz) band for HRV and BOLD signals in relation to interoceptive perception^10^ and by the strong information flow from cortex to brainstem observed during elevated fMRI-related in the IMF (0.1–0.2 Hz) band. Besides oscillations in blood pressure with frequencies below 0.1 Hz^43^, the cardiovascular rhythm in the 0.15-Hz band with an origin in the reticular formation in the brainstem^11^, and BOLD oscillations between 0.13 and 0.17 Hz^44^, coherent breathing and RRI oscillations at ~ 0.15 Hz have also been reported during low and elevated anxiety^33^. A wave-by-wave analysis in a few healthy subjects revealed a breathing period duration of 6.7 ± 0.33 s (mean ± SD) and a RRI period duration of 6.6 ± 0.29 s. This was associated with a coherence between the two rhythms, which is characteristic for negative RSA^34, 35^.

While the center frequency of slow BOLD oscillations at 0.08 Hz with an origin in the baroreflex loop is well documented and in accordance with the recommended lowpass filtering at 0.1 or 0.09 Hz for standard resting state fMRI studies^52, 53^, the situation of slow frequency oscillations above 0.1 Hz needs discussion. On the one hand, there are reports about oscillations centered at ~ 0.15 Hz in neural activity, respiration and cardiac signals^11, 33, 44, 54^, but on the other hand, 0.08-Hz oscillations often have a non-sinusoidal form and, therefore, are accompanied by harmonic components centered at 0.16 Hz. Note, that the model of Klimesch^36, 37^ concerning coupling of EEG and body rhythms includes 0.08 Hz and 0.16 Hz oscillations. When considering the well-known 0.15-Hz rhythm in the brainstem^11^ and the model of Klimesch^36, 37^ with the coupled 0.08 and 0.16 Hz oscillations to reduce the energy demand, we recommend to use the term “0.15/0.16 Hz” for the dominant oscillations in the 0.1–0.2 Hz band.

**Figure 5:**
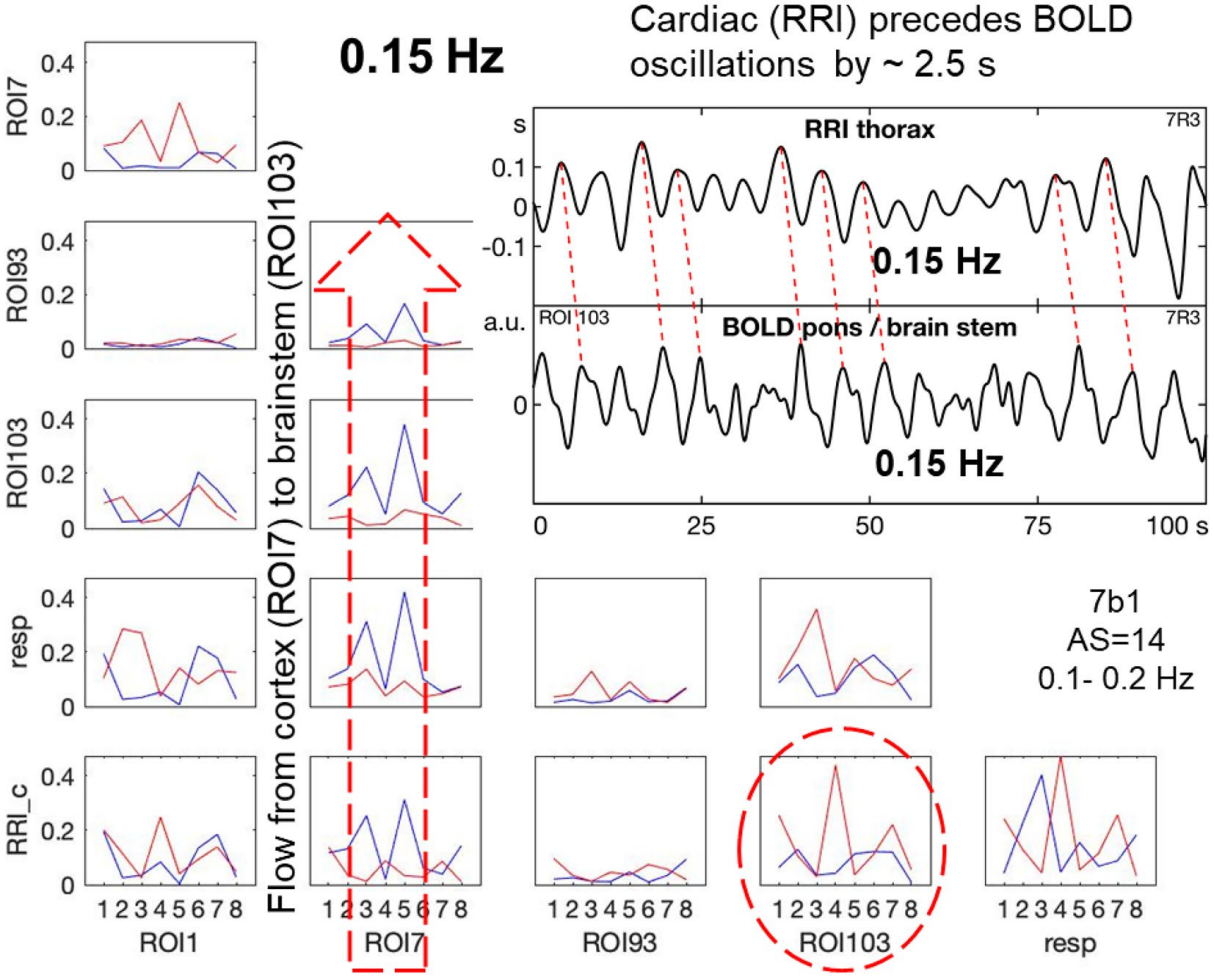
Example of a subject (7R3) with a stable, coherent resting state 0.15-Hz oscillation in RRI, respiration and BOLD signals in the cortex and brainstem during low anxiety (AS = 14). In each box ffDTF as a function of consecutive eight time windows of 40 s length is shown. Blue lines: outflow from the structure marked below the box to the structure marked at the left, red lines: flow from structure marked at the left to this marked below. The insert (top right) shows time courses of signals: RRI (upper curve) and BOLD from ROI 103 (lower curve). Note that RRI precedes BOLD oscillations by ~ 2.5 s (insert) and that a strong information flow can be observed, going from the cortex (MFG, ROI 7) to the brainstem (ROI 103) as marked by the stippled arrow.

Relatively stable ongoing oscillations at 0.15/0.16 Hz in RRI, breathing and BOLD signals are rare and were found only in one participant with dominant neural BOLD oscillations and low state anxiety (AS = 14; due to this low anxiety not included in the HA11 group). Remarkable is the dominant information flow from the cortex (MFG) to the brainstem including respiration and RRI (stippled arrow in Fig. 5). Examples of a raw BOLD signal in the brainstem (ROI 103) and the RRI signal (shown in the insert of Fig. 5) clearly depict oscillations in both signals, the former lagging behind the RRI signal in an interval that reflects neurovascular coupling time. This lag corresponds to an information flow from RRI to the associated BOLD response in the brainstem (stippled ellipse in Fig. 5).

This example, as shown in Fig. 5 is of special interest because of the coherent oscillations in cardiac, respiratory and BOLD signals at ~ 0.15 Hz and the strong information flow from the cortex to the brainstem. In this specific case of coherent signals, a synchronizing source is very likely. Such a source can be an externally paced stimulus for inspiration in intervals close to the resonance frequency of the cardiovascular system^38, 39^ but also a pacemaker-like activity in the form of an autonomous oscillator in the brainstem. In the latter case, an ascending information flow from the brainstem to the cortex might be expected (example see Fig. 3), but as Fig. 5 indicates, this is not the case. Whether this case represents an example of resonance between the cardiovascular and the respiratory system at a frequency close to 0.16 Hz, which would be consistent with the model of Klimesch^36, 37^ is on open question, which needs further research.

State anxiety can be evaluated by a questionnaire while lying with the head in the scanner^14, 18^ but also while sitting on a chair in a quiet room and wearing a facemask for measuring respiratory pattern and metabolism^55^. In both cases a differentiation between low and high anxiety is possible, although major anxiety differences are expected in the scanner, but not during sitting in a quiet room. Nevertheless, Kato et al.^55^ reported in a study with young men a significant increase of the respiration rate from 12 to 17 breaths/min with higher anxiety, compared with lower anxiety. This example provides evidence, that neurons driving the breathing in the brainstem at frequencies in the IMF band are most sensitive to negative emotions. Although the spectrum of anxiety evaluated by a questionnaire^19^ is substantial and varies from “bad gut feeling” (interoception) to abortion due to claustrophobia, an fMRI examination without any indications of anxiety is very unlikely.

### Upward information flow into the precentral gyrus during prevailing anxiety

The precentral gyrus (PCG) is called the somatomotor cortex because it controls volitional movements and is a very important part of the large-scale somatomotor network (SMN). Dysfunction in the SMN has been reported in various neuropsychiatric disorders and in anxiety disorders (AD). Tumati et al.^9^ reported on abnormal functional connectivity primarily in SMN and default mode network (DMN). Namely, the functional connectivity is increased in SMN and decreased in DMN in AD. It can be speculated that the increased flow into the PCG is related to the increased connectivity not only in AD, but also in healthy subjects during elevated fMRI-related anxiety. This speculative relationship is of interest, but needs further research.

This relatively clear functional assignment of the nearby located ROIs 93 and 103 in pontine structures (ROI 103 related to activation of neurons in cardiovascular and respiratory centers and ROI 93 related to activation of pacemaker-like source in brainstem) excludes largely that the BOLD signals in brainstem represent physiological noise or other artefacts^56^. A short comment is necessary concerning the use of the LF band. Although recommended by the Task Force^13^, the upper limit of the band at 0.15 Hz coincides with the cardiovascular 0.15 Hz rhythm of Perlitz et al.^11^, with the center frequency at 0.16 Hz of the frequency architecture of brain-body oscillations^36, 37^, and with our findings of coupling between cortex and brainstem. Therefore, the LF band is perhaps optimal for all types of HRV studies^57^, but is not best suitable to study brain–heart interaction and fMRI-related anxiety.

### RRI, respiratory and BOLD signals in brainstem

In Fig. 4 it is shown that during prevailing or elevated anxiety the information flow is from BOLD signal in pons/brainstem to the prefrontal cortex and to the RRI signal, meaning the rhythmic neural source (pacemaker) operating in the frequency range 0.1–0.2 Hz “drives” the RRI oscillations. What is not shown in Fig. 4 is, that this pacemaker may also stimulate one of the respiratory centers either 1:1, 1:2 or 1:3 (one, two or three breaths during one cardiac wave). In the case of the 1:1 ratio the cardiovascular and respiratory systems are synchronized, characterized by one dominant frequency, negative RSA (nRSA)^33, 34^ and reduced energy demand. This is a situation observed in scanner in ~ 20% of participants with elevated fMRI-related anxiety. Ratios of 1:2 or 1:3 are also found during RSA and nRSA, similarly representing a form of synchronization between physiological systems. Note, beside the binary hierarchical model of Klimesch^36, 37^, there also exists 1:3 coupling. Further research work is necessary to clarify the complex interaction between various BOLD oscillations in brainstem and physiological signals with focus on nasal and oral breathing.

## Conclusions

The obtained results can be summarized as follows: (i) During fMRI-related anxiety, either low or elevated, different coupling patterns with predominant oscillations in the IMF band centered at 0.15/0.16 Hz can be observed. During low/no anxiety the information flow from cortex to brainstem is dominant, while during elevated anxiety the flow with an origin in the brainstem to the cortex dominates. (ii) During elevated anxiety the intensified information flow from cortex and from brainstem modulates the cardiac interval (RRI) signal. This observation provides further evidence for the localization of an activated neural source in the brainstem (pacemaker). (iii) The placement of the head in a noisy, constricted space within the scanner is always accompanied by some level of anxiety, starting with uneasy feeling and ending in claustrophobia. Noteworthy, four healthy participants (~ 20%) displayed an unexpected growth of fMRI-related anxiety with high anxiety scores at the end of scanning. Inspection of the data of these persons revealed some special features, namely all four displayed neural BOLD oscillations (pacemaker in brainstem), a prominent respiration at ~ 0.33 Hz and an information flow from slow cardiac to respiratory oscillations (negative RSA^33, 34^). This impact of anxiety, either low or high, on brain and body oscillations during scanning should be considered in each scientific fMRI study.

A novel result is the intensified flow centered at 0.15/0.16 Hz from cortex to brainstem during low or no anxiety. The existence of such frequency components in resting state networks is not only supported by the finding of BOLD oscillations in the 0.13–0.17 range^44^ but also by the high correlation between HRV and BOLD signals in the 0.12–0.18 Hz band^10^. In the phase coupling model of Klimesch^36, 37^ for EEG and body oscillations, the 0.16 Hz represents, besides 0.08 Hz (halving of 0.16 Hz) and 0.32 Hz (doubling of 0.16 Hz), an important center frequency.
